# Targeting microRNA-143 in colorectal cancer: advances in molecular biosciences for biomarker-based diagnostics, therapeutic strategies, and drug resistance prediction

**DOI:** 10.3389/fmolb.2025.1679533

**Published:** 2025-12-10

**Authors:** Xue Wang, Lei Wang, Long Ke

**Affiliations:** 1 Department of Oncology, Mianyang Fulin Hospital, Mianyang Fulin Hospital Co., Ltd., Mianyang, China; 2 Second Department of Gastrocolorectal Surgery, Jilin Cancer Hospital, Changchun, Jilin, China; 3 Key Laboratory of Gastrointestinal Tumor Bioinformatics of Jilin Province, The first Hospital of Jilin University, Changchun, Jilin, China; 4 Department of Health Examination, The Third Affiliated Hospital of Shanghai University, Wenzhou No.3 Clinical Institute Affiliated to Wenzhou Medical University, Wenzhou People’s Hospital, Wenzhou, China

**Keywords:** mir-143, colorectal cancer, non-coding RNAs, molecular diagnostics, drug resistance

## Abstract

Colorectal cancer (CRC) persists as a significant global health challenge, distinguished by intricate molecular modifications and a notable propensity for resistance to standard therapeutic interventions. Among the regulatory factors contributing to CRC pathogenesis, microRNAs (miRNAs) have emerged as pivotal regulators of gene expression, presenting innovative prospects for diagnostic and therapeutic advancements. Notably, microRNA-143 (miR-143) has attracted considerable attention as a tumor-suppressive miRNA, exhibiting diverse functions in the development, progression, and therapeutic response of CRC. This review delineates an exhaustive examination of the molecular mechanisms by which miR-143 modulates critical oncogenic pathways, encompassing KRAS signaling, epithelial-mesenchymal transition, and metabolic reprogramming. We underscore recent progress in the molecular biosciences that position miR-143 as a promising biomarker for the early detection and prognosis of CRC. Furthermore, we investigate its emergent function in the modulation of sensitivity to chemotherapeutic and targeted therapeutic agents, emphasizing its potential utility in predicting and mitigating drug resistance in CRC cells. By synthesizing contemporary findings within the domains of molecular diagnostics and therapeutic interventions, this review accentuates the clinical potential of targeting miR-143 in the personalized management of CRC and the prediction of drug resistance.

## Introduction

Approximately 10% of global cancer cases and deaths are ascribed to colorectal cancer (CRC), rendering it the second most prevalent cancer in women and the third most prevalent in men. As developing countries continue to make advances, the global rate of CRC cancer is expected to rise to 2.5 million new cases by 2035 (Bray et al., 2018; Dekker et al., 2019; Li et al., 2021; Thanikachalam and Khan, 2019). Epidemiological research consistently identifies advancing age and male sex as significant risk factors for CRC, though its progression is driven by a complex interplay of hereditary traits and environmental elements.

The majority of CRCs develop from pre-cancerous polyps through a well-defined histopathological process known as the adenoma-carcinoma sequence (Pei et al., 2024; Li et al., 2024). This process typically initiates with an abnormal crypt that develops into a benign adenomatous polyp. Over 10–15 years, the progressive accumulation of genetic and epigenetic alterations—such as mutations in *APC*, *KRAS*, and *TP53*—drives the transformation of this polyp into an invasive CRC (Xu et al., 2023; Tong et al., 2022). This multistep progression involves both the dysfunction of tumor-suppressing genes and the activation of oncogenes. Furthermore, this process is increasingly understood to be driven by a subpopulation of cells with stem-like characteristics, or “cancer stem cells,” which are key players in tumor initiation, maintenance, and therapeutic resistance (Li et al., 2021; Chu et al., 2024; Batlle and Clevers, 2017; Lathia et al., 2015).

MiRNAs, a type of short non-coding RNA ranging from 18 to 26 nucleotides, are synthesized in the body as a natural process. These tiny molecules can communicate with numerous mRNAs, playing a crucial role in a vast and intricate regulatory system (Sadri Nahand et al., 2022; Davoodvandi et al., 2023a). They involve in regulating various cellular activities (Davoodvandi et al., 2023b).

While numerous primary studies have investigated miR-143, these findings often remain fragmented, focusing on singular pathways (e.g., KRAS regulation, EMT) or functions. The specific novelty and purpose of this review is to provide a comprehensive, integrative synthesis that bridges this gap between mechanism and application. We amalgamate the dispersed research into a cohesive framework, explicitly connecting miR-143s molecular functions to its translational potential in three key areas: its utility in molecular diagnostics, its potential as a therapeutic target, and its critical role in predicting and modulating drug resistance.

## MicroRNA biogenesis and function

The fundamental rules and strategies for regulation and targeting were already evident through molecular genetic research in *Drosophila* (Leviten et al., 1997), Before miRNAs were found in that particular species, they were already recognized as a crucial group of small RNAs present in more complex organisms in 2001. This discovery showed the importance of miRNAs as important regulators different cell function (Lagos-Quintana et al., 2001). Currently, numerous miRNAs were identified in diverse organisms, with their main function being the binding to mRNA and subsequently causing its degradation, which permits the control of both gene expression and protein production.

**FIGURE 1:**
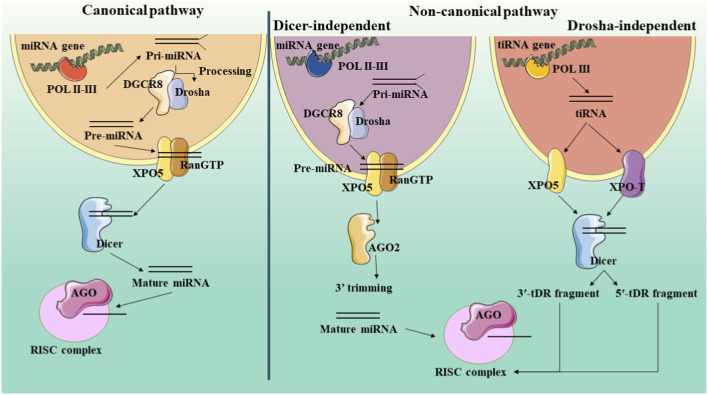
Schematic representation of microRNA biogenesis pathways. This figure illustrates the canonical and non-canonical pathways for microRNA (miRNA) biogenesis. Canonical Pathway (Left): A miRNA gene is transcribed by POL II into a pri-miRNA. This is processed in the nucleus by the Drosha-DGCR8 microprocessor complex into a pre-miRNA. The pre-miRNA is exported to the cytoplasm by XPO5/RanGTP. In the cytoplasm, Dicer cleaves the hairpin into a mature miRNA duplex. This duplex is loaded into an Argonaute (AGO) protein, where the passenger strand is discarded, and the mature miRNA guide strand, now part of the RISC complex, targets mRNA for silencing. Non-canonical Pathways (Right): These pathways bypass either Drosha (e.g., Mirtrons, which are spliced introns) or Dicer (e.g., some shRNAs processed directly by AGO2). tiRNAs are shown as an example of a Drosha-independent pathway, where tRNAs are cleaved by Dicer into tDR fragments that can be loaded into RISC.

As previously stated, miRNAs are composed of 22 nucleotides and are derived from pri-miRNA transcripts, which have hairpin structures and can vary in length. Most miRNA hairpins originate from non-coding or intronic transcripts, while a minority are found within exons of protein-coding genes. There exist two processes through which miRNAs can be created: the non-canonical pathways and canonical (both of which are illustrated in detail in Figure 1). Although the majority of conserved miRNAs are believed to be produced through the canonical pathway.

### Canonical pathway

In the canonical pathway, the initial phase entails the function of Pol II, which is accountable for transcribing specific miRNA genes. Around 30% of these miRNAs originate from the introns of genes that code for proteins, while others are formed from specific locations of miRNA genes. The consequence of this process is the creation of a pri-miRNA, which then requires further modifications such as capping, splicing, and polyadenylation. The microprocessor complex, consisting of the RNA binding protein DGCR8 and the ribonuclease III enzyme Drosha, facilitates the production of pre-miRNA (Denli et al., 2004). Aside from Drosha, this complex of multiple proteins comprises Pasha, which is Drosha’s companion and a protein responsible for binding to double-stranded RNA. When the expression of Pasha is inhibited in cells of *Drosophila* or *Caenorhabditis elegans*, it hinders the conversion of pri-miRNA, causing a rise in the levels of pri-miRNAs and a decline in mature miRNAs. (Denli et al., 2004). It has been determined through research that the functions of these proteins involve the recognition of the connection between the stem and adjacent single-stranded RNA of the pri-miRNA hairpin (by DGCR8) and the cleavage of the RNA duplex to generate the pre-miRNA product (by Drosha) (Alarcón et al., 2015). After being produced, pre-miRNAs that have a 2-nucleotide overhang at the 3′end are moved to the cytoplasm with the help of XPO5 and the RanGTP complex. Thereafter, the RNase III endonuclease Dicer is responsible for their modification (Okada et al., 2009). Finally, Dicer generates a mature form of miRNA that requires binding to the RISC, an effector ribonucleoprotein complex, to carry out its functions (Yoda et al., 2010). Despite the growing body of research in this area, the exact components of the RISC complex remain somewhat unclear to usIt is evident, however, However, a vital element is a protein belonging to the Argonaute (Ago) family, which plays a pivotal role in the formation of RISC. The Ago family of proteins can be segregated into two categories: One is found in various body parts (AGO subfamily) and another is only found in reproductive organs (PIWI subfamily proteins). In *Drosophila*, there are two AGO proteins known as Ago1 and Ago2, while humans possess four, known as Ago1 through Ago4 (Hutvagner and Simard, 2008; Siomi and Siomi, 2009). After forming a double-stranded intermediate by Dicer, this intermediate is loaded into AGO proteins in order to form protein complexes known as pre-RISCs contain small-RNA duplexes. The variability in the ratio of 5p or 3p strands loaded with AGO for a particular miRNA greatly depends on the type of cell or the surrounding environment, which can result in almost equal proportions or a strong bias towards one specific strand (Meijer et al., 2014). The influence on the decision between the 5p and 3p strand is partially derived from the thermodynamic stability found at the 5′ends of the miRNA double helix. This is specifically seen when the first nucleotide position possesses a 5′uracil. In most cases, the weaker stability at the 5′end or the presence of a 5′uracil leads to the preference for the guide strand to be inserted into AGO. The remaining strand, known as the passenger strand, will then undergo various processes to be separated from the guide strand, depending on their level of complementarity (Meijer et al., 2014). To avoid any errors, the incorrect miRNA passenger strands are specifically targeted and cut by the enzyme AGO2. The cellular processes within the body then break down these strands, leading to a significant favoritism towards one particular strand. However, miRNA double-stranded structures containing mismatches at their core or lacking AGO2 loading are subjected to passive unwinding and subsequent degradation (Meijer et al., 2014).

### Non-canonical pathways

Despite the variations between them, there are particular similarities between canonical and non-canonical pathways. Specifically, Dicer, exportin 5, AGO2, and Drosha are all shared components. In broad terms, methods of miRNA production that deviate from the typical route can be categorized into those that do not involve Drosha/DGCR8 and those that do not require Dicer. A well-known example of the former is the creation of mirtrons from mRNA introns during the splicing process. Additionally, unconventional handling of transfer RNAs (tRNAs) also contributes to the production of miRNAs derived from tRNAs. After transcription, tRNAs are transported to the cytoplasm through either XPO-5 or XPO-T, where they are subsequently cleaved by the Dicer enzyme, resulting in the formation of 5′-tRNA-derived RNA (tDR) fragments from the 5′-loop and 3′-tDR fragments from the 3′-loop. Moreover, ANG has the ability to cut the anticodon loop, resulting in the production of tiRNAs, shorter fragments of tRNA that are caused by cellular stress. These tiRNAs are then loaded into RISC, similar to canonical miRNAs. Unlike conventional miRNAs, which are produced by Dicer, a class of miRNAs called Dicer-independent miRNAs are created by the enzyme Drosha from internal shRNA molecules (Yang et al., 2010). Due to their insufficient length for Dicer processing, these pre-miRNAs rely on AGO2 in the cytoplasm for their full maturation. This is accomplished by the pre-miRNA being fully integrated into AGO2, allowing for the 3p strand to be sliced off. The final step of maturation involves trimming the 5p strand in a 3′to 5′ direction (Yang et al., 2010; Cheloufi et al., 2010).

### Molecular mechanisms of miR-143 in CRC

**FIGURE 2:**
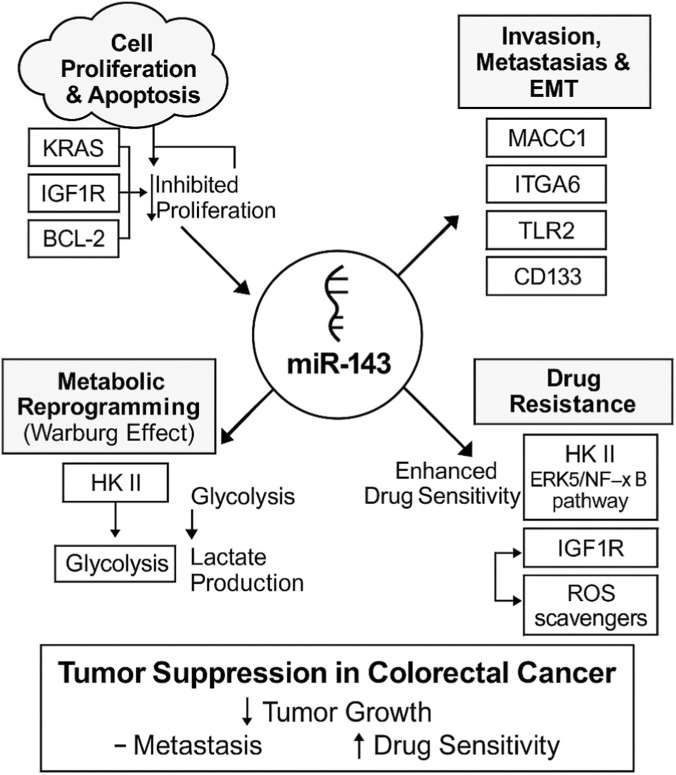
Multifaceted tumor-suppressive roles of miR-143 in colorectal cancer (CRC). This schematic illustrates how miR-143 acts as a central tumor suppressor by modulating various oncogenic pathways and processes in CRC. The arrows from miR-143 indicate its regulatory impact on each process. Cell Proliferation and Apoptosis: miR-143 inhibits proliferation. This process is shown to be influenced by KRAS, IGF1R, and the anti-apoptotic protein BCL-2. Invasion, Metastasis, and EMT: miR-143 suppresses these processes by targeting key regulators, including MACC1, ITGA6, TLR2, and CD133. Metabolic Reprogramming: miR-143 inhibits the Warburg effect by targeting Hexokinase II (HK II), which in turn reduces glycolysis and lactate production. Drug Resistance: miR-143 enhances drug sensitivity by targeting multiple resistance pathways, including HK II, the ERK5/NF-κB pathway, IGF1R, and ROS scavengers.

According to accumulative studies, the expression of mir-143 has an association with different aspects of CRC including pathohistological changes, initiation, proliferation, apoptosis, invasion, and metastasis which are explained in this section in details (Figure 2).

### Inhibiting invasion and metastasis

**FIGURE 3:**
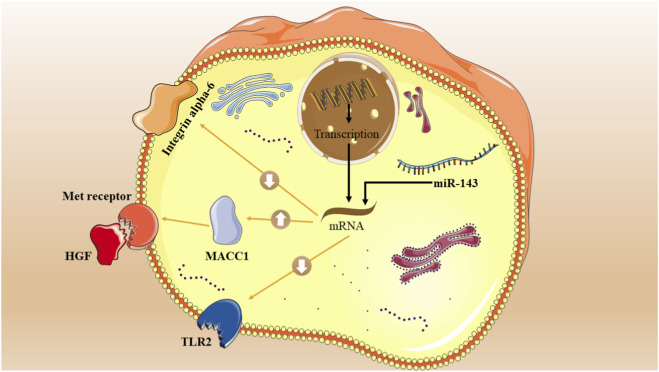
Molecular mechanisms by which miR-143 affects invasion, migration, and metastasis of CRC cells. This diagram depicts the tumor-suppressive roles of miR-143 in CRC. In the nucleus, genes are transcribed into mRNA. In the cytoplasm, the mature miR-143 (blue strand) binds to target mRNAs, leading to their degradation (represented by the black blunt-ended arrow) and preventing their translation into protein. Inhibition of ITGA6: miR-143 targets ITGA6 (Integrin alpha-6) mRNA, reducing its protein expression (indicated by the downward arrow) to inhibit cell adhesion and migration. Inhibition of MACC1: miR-143 targets MACC1 (Metastasis-associated in colon cancer 1) mRNA, inhibiting its translation. This blocks MACC1’s ability to promote the HGF/Met pathway (shown separately), thereby inhibiting invasion. Inhibition of TLR2: miR-143 targets TLR2 (Toll-like receptor 2) mRNA, reducing its protein expression (downward arrow) to suppress inflammatory pathways that promote migration. By inhibiting these key targets, miR-143 collectively reduces CRC cell invasion and metastasis.

In 2010, Kulda et al. found that miR-143 disruption affects late-stage CRC cell metastasis and invasion showed Figure 3 (Kulda et al., 2010). This study analyzed the correlation between miR-143 levels and important clinical and pathological factors of CRC and CLM. CLM tissue had significantly lower miR-143 expression than healthy colon tissue. Additionally, a marked increase in miR-143 expression was detected in CLM tissue compared to non-cancerous liver tissue (Kulda et al., 2010).

Integrin alpha-6, involved in cell adhesion and signalling, consists of an alpha chain and a beta chain, and is encoded by the ITGA6 gene. This cell-surface protein, also known as VLA-6, ITGA6, or CD49f, is responsible for facilitating cellular adhesion with both other cells and the surrounding stroma. The importance of this adhesion is critical for cell growth, movement, survival, and development (Khademi et al., 2023). It was demonstrated by a study on 6 patients with CRC and liver metastasis that the migratory and invasive capabilities of LoVo cells considerably reduced 24 h after they were transfected with miR-143-3p mimics. This decline can be attributed to the targeting of the ITGA6 gene by the miRNA, as observed in the control groups (Guo et al., 2019). At a concentration of 100 nmol of transfected miR-143-3p mimics, ITGA6 mRNA expression decreased by 4.5 times. Furthermore, they also detected that the mRNA expression of Ankyrin repeat and PH domain 3 (AGAP3) (which is a gene that produces a protein capable of binding phosphoinositides) fell to over five times lower than that in the control groups (Guo et al., 2019).

Recent research has identified a gene called MACC1 that plays a significant role in the progression and spread of CRC. This gene works closely with the HGF/Met signaling pathway to stimulate the growth, invasion, and dispersion of CRC cells within their surrounding environment, leading to increased metastasis and tumor growth in animal models. The activation of MACC1 is a crucial element in this process (Stein et al., 2009). In regards to this, an attempt was made by Zhang et al. to establish a connection between the functions of MACC1 and how miR-143 may reduce CRC cell metastasis and invasion (Zhang et al., 2012). The identification of MACC1 as a target of tumor-suppressing miR-143 in CRC was established via *in silico* prediction and a Western blot assay. Their direct interaction was validated using a 3′UTR luciferase reporter gene. The re-introduction of miR-143 mimics into SW620 cells significantly reduced invasion, cell growth, and migration, consistent with the findings from MACC1 knockdown via siRNA [48]. Furthermore, an inverse relationship was found between miR-143 levels and MACC1 mRNA expression in CRC tissues, further affirming the tumor-suppressing function of miR-143 in this particular cancer (Zhang et al., 2012).

The external cellular membrane contains the Toll-like receptor 2 (TLR2), a member of the TLR superfamily. It has the capability to initiate the MyD88-dependent canonical pathway through the recruitment of the IRAK complex and TRAF, which activates the MAPK, NF-κB, and AP-1. Cytokines that promote inflammation are produced as a consequence of this process (Di Lorenzo et al., 2020). Guo et al. (2013) examined tissues from patients with CRC and observed an increase in the levels of TLR2 in these samples. They also declared that by targeting TLR2, the invasion and migration of CRC cells are substantially reduced (Guo et al., 2013). It was discovered that the invasion and migration of CRC cells through TLR2 is reduced by miR-143. Based on a xenograft mouse model, it was observed that the inhibition of CRC cell colonization *in vivo* is achieved through the re-expression of miR-143 (Guo et al., 2013).

### Reducing proliferation and apoptosis

Another essential effect of mir-143 on CRC cells is how it can affect cell cycle and thereby, regulate proliferation and apoptosis of these cells (mentioned studies are summarized in Table 1). A notable decrease in the expression levels of mir-143 in SW-480 human CRC cells has been observed by Yang et al., leading to a noteworthy increase in proliferation 48, 72, and 96 h following transfection with said RNA (Yang et al., 2015). When 480 cells were transfected with miR-143, flow cytometry revealed a significant decrease in the proportion of cells in the S phase and an increase in the proportion of cells in the G1 phase (Yang et al., 2015). On the other hand, it is important to point out that the regulation of the cell cycle has been discovered to result in an increase in the rate of apoptosis in these cells (Yang et al., 2015). Paradoxically, another study demonstrated that miR-143 was able to hinder the growth of cells *in vitro* (Akao et al., 2010). They also demonstrated that the resistance to nuclease is increased and the levels of miRNA are five to eight times greater when chemically modifying miR-143 through the incorporation of 30-benzen-pyridine (BP) at the 30-overhang region, known as miR-143BP, instead of using miR-143 from Applied Biosystems (Akao et al., 2010). Furthermore, Akao et al. corroborated the inhibitory impact of this particular miRNA on tumors in an animal model. They conducted a study in which three sets of mice were given varying doses (high: 50 mg per mouse, low: 25 mg per mouse) of miR-143BP through intravenous injections once a week for 5 weeks. The mice were required to have a tumor volume of at least 300 mm3 before the injections could commence on day 14. The mice were euthanized and their tumor weights measured to assess the effectiveness of the therapy. Results showed a significant decrease in tumor weight in the miR-143BP group compared to the control group, starting from 3 weeks after the injections (Akao et al., 2010). Numerous studies have revealed that tumors frequently display an excessive amount of the Insulin-like Growth Factor 1 Receptor (IGF1R), which is a type of enzyme receptor that binds to IGF-1 and IGF-2. Through extensive investigations using human samples, cell cultures, and animal models, this receptor is essential in numerous significant processes, including the development of malignant cells, advancement of tumors, metastasis, and avoidance of cell death (Riedemann and Macaulay, 2006). There are three transmembrane proteins that make up the group of proteins that are referred to as IGF receptors. The IGF1R gene is situated on chromosome 15q26. This gene is responsible for coding a singular polypeptide composed of 1,367 amino acids, which is consistently present in the vast majority of cells (Riedemann and Macaulay, 2006). Su and colleagues investigated the relation between IGF1R gene and mir-143 (Su et al., 2014). Using bioinformatics, researchers were able to discover the locations of miR-143 target sites in the 3′-UTR of the IGF1R gene. They found that the energy required for the binding between miR-143 and IGF1R was −19.8 kcal/mol, indicating that they form a legitimate miRNA-target pair. Additionally, the specific binding sequences of miR-143 in the IGF1R 3′-UTR were found to be highly similar among various species, demonstrating a significant level of conservation (Su et al., 2014). After measuring miR-143/145 and IGF1R protein levels in six sets of CRC tissues and their noncancerous counterparts, miR-145 and miR-143 were consistently lower in CRC tissues. In contrast, the levels of IGF1R protein were amplified in the CRC tissues. The findings indicate an inverse correlation between the expression levels of miR-143/145 and IGF1R protein in human CRC tissues (Su et al., 2014). The examination of IGF1R mRNA expression was conducted after transfection and showed that IGF1R expression is directly regulated by miR-143 at the post-transcriptional level (Su et al., 2014). Finally, they detected that the increased levels of IGF1R significantly countered the inhibitory impact of miR-143 on cell growth (Su et al., 2014). A separate study demonstrated that down-regulating this microRNA could enhance the viability of CRC cells. Chemotherapeutic drugs, such as crocin, have the potential to counteract this effect (Hosseini et al., 2023). It was demonstrated by this study that crocin administration can lower the expression of KRAS and RREB1, and lead to the dephosphorylation of AKT, all of which are likely due to the upregulation of miR-143 (Hosseini et al., 2023). Karimi et al. (2019) used another method and achieved the restoration of miR-143 by introducing the pCMV-miR-143 vector into the SW-480 CRC cells through transfection. After the analysis of the mRNA levels of certain genes, it was discovered that cells exposed to miR-143 demonstrated lowered viability and migratory capacity. Apoptosis was confirmed using DAPI staining. Additionally, lower levels of K-RAS, MMP-9, c-MYC, and BCL-2 mRNAs were observed in cells treated with miR-143. The ratio of BAX to BCL-2 was found to be significantly higher in cells with elevated levels of miR-143. Overall, replacing miR-143 shows promise in targeting CRC and reducing its invasive properties (Karimi et al., 2019).

**TABLE 1:** Studies investigating the effects of mir-143 on inhibiting colorectal cancer cell proliferation.

Author(s)	Publish year	Model	Result(s)	Reference
Yang et al.	2015	*In vitro*	• ↑miR-143 → ↓proliferation (S-phase arrest) • ↑miR-143 → ↑apoptosis in SW-480 cells	Yang et al. (2015)
Akao et al.	2010	*In vitro* & *In vivo*	• Inhibited CRC cell growth *in vitro* • Chemically modified miR-143BP → ↓tumor weight *in vivo*	Akao et al. (2010)
Su et al.	2014	*In vitro*	• Suppresses proliferation by directly targeting IGF1R	Su et al. (2014)
Hosseini et al.	2023	*In vitro*	• Crocin → ↑miR-143 • ↑miR-143 → ↓proliferation via KRAS/RREB1 pathway	Hosseini et al. (2023)
Karimi et al.	2019	*In vitro*	• miR-143 replacement → ↓viability & ↑apoptosis • Downregulated BCL-2, K-RAS, c-MYC	Karimi et al. (2019)

The strongest lines of evidence in this domain converge on miR-143s ability to directly target key oncogenic drivers like KRAS and IGF1R, and metastatic promoters like MACC1. These findings, replicated across numerous *in vitro* and *in vivo* models, provide a solid mechanistic basis for its tumor-suppressive role. A remaining gap, however, is understanding whether this suppression is equally effective across all CRC molecular subtypes (e.g., CMS1-CMS4), which may have different dependencies on these pathways.

### Diagnostic and prognostic roles of miR-143

#### miR-143 as a prognostic marker

Other than therapeutic applications, microRNAs can also be used for prognostic purposes and mir-143 is not an exception. With respect to this, Li and colleagues performed a meta-analysis for assessing the accuracy of using this microRNA for determining CRC prognosis. They found a total of 5,128 patients from 17 articles in this field (Li et al., 2019). A study examining the tissue of 2,472 individuals with CRC found no significant association between low levels of miR-143 and reduced overall survival. However, when examining the link between miR-143 and event-free survival, miR-143 was found to be the most reliable predictor. Simmer et al. also looked at the correlation between 22 microRNAs and PFS, discovering that miR-143 expression had the strongest impact on PFS. Notably, individuals with diminished levels of miR-143 exhibited an extended duration of PFS (Simmer et al., 2015).

### The relation between mir-143 and clinical findings of CRC

Investigating the relation between this microRNA and histological and clinical findings in CRC can help Another study on human samples of CRC also showed that among all dysregulated microRNAs, miR-143 expression is also remarkably lower in different ages, CRC grades, and tumor depth and sizes (Akao et al., 2010). For instance, among 45 male samples in this study 75.6% were found to have lower mir-143 levels while among 18 female samples, 61.1% had lower mir-143 levels (Akao et al., 2010). In different clinical stages of CRC, it seems that mir-143 expression is reduced in 75%–80% of cases of I, IIIa, IIIb, and IV while only 57.9% of cases with II stage were detected with a reduction in their mir-143 levels (Akao et al., 2010). Furthermore, mir-143 reduction was mostly observed in tumors which had the depth of Subserosa and Mucosa propria (Akao et al., 2010). However, the expression of this microRNA in different sites of tumor is not very different (74.5% and 70% of proximal and distal colon cancers have low mir-143 levels, respectively). On the contrary, Akao et al. showed that tumors with a diameter of more than 50 mm are more likely to have reduced mir-143 in comparison to smaller tumors (Akao et al., 2010). In addition to these findings, they examined the impact of co-expressing miR-143 and miR-145 on the proliferation of DLD-1 cells. This combination treatment consisted of delivering miR-143 and miR-145 at 20 nM each, which was half the dose used when individually administering miR-143 (40 nM) or miR-145 (40 nM) (Akao et al., 2010). An additive effect was exhibited when a treatment of 20 nM each of miR-143 and -145 was utilized, resulting in approximately 74% and 78% growth inhibition, respectively, in comparison to the control (data not presented) (Akao et al., 2010).

### miR-143 expression in colorectal adenomas

Akao et al. are among the groups that attempted to ascertain the levels of miR-143 in colorectal adenomas. They detected that the same as CRC cells, the reduction of this miRNA is seen in males more than females. Plus, 73.8% of adenomas of proximal colon have a reduced miR-143 while only 52.2% of distal colon adenomas show this characteristic. They also observed that adenomas with the diameter of 10 mm or more are more likely to have reduced miR-143 levels (Akao et al., 2010). Furthermore, adenomas with the exophytic shape have a 17% higher possibility of having reduced miR-143 levels (Akao et al., 2010). In 2019, an attempt was made to establish a connection between miR-143 expression and colorectal adenomas in a study. A study was carried out on 45 individuals diagnosed with CRC, specifically 24 cases of colon cancer and 21 cases of rectal cancer. Additionally, the study analyzed 11 advanced adenomas, 14 hyperplastic polyps, and 48 control subjects. This research was part of a larger case-control study conducted at various hospitals in Colombia (Ardila et al., 2019). The quantities of various microRNAs in tissue samples from the referenced patients were analyzed by the researchers. Significantly higher miR-143-3p levels were observed in the adenoma group compared to the control group (Ardila et al., 2019). In 2021, a study was conducted by researchers led by Yamano to investigate the unique circulating miRNAs in individuals with FAP, a hereditary digestive disorder. The results revealed that miR-143-3p levels were elevated in FAP patients compared to healthy donors (P = 0.04), although the impact of clinical and pathological characteristics was not significant. Additionally, while miR-143-3p upregulation was not commonly observed in colonic tumors, notable variations were observed in the presence of desmoid tumors. Furthermore, the transfection of miR-143-3p led to a notable suppression of CRC cell proliferation compared to the transfection of a control microRNA (Yamano et al., 2021). Their result revealed that the regulation of miR-143-3p expression in most FAP patients differed based on the type of sample, whether from plasma or colonic tumors. Detecting higher levels of plasma miR-143-3p could potentially help diagnose FAP (Yamano et al., 2021).

The strongest evidence for miR-143 as a biomarker is its consistent downregulation in CRC tissues compared to adjacent normal mucosa, a finding that holds true from early adenomas to late-stage carcinomas. However, its prognostic role presents a significant controversy. While most studies link low miR-143 to poor outcomes, the study by Simmer et al. (2015) found that *diminished* miR-143 levels correlated with *longer* PFS in patients on capecitabine. This apparent contradiction highlights a critical gap in knowledge: miR-143s function may be context-dependent, potentially shifting from a general tumor suppressor to a specific modulator of drug metabolism, warranting further investigation.

### Therapeutic implications of miR-143

#### Modulating drug resistance in CRC cells

Drug resistance is another essential factor for predicting the outcomes of treatment and estimating the overall survival. In the context of CRC, there are several therapeutic options for metastatic and non-metastatic cancers. All healthy stage III colon cancer patients should receive chemotherapy after tumor removal. Adjuvant chemotherapy should be considered for evaluation in all cases of clinical and/or histological stage II and III rectal cancers, as well as for those patients with high-risk characteristics including T4 tumors, tumor perforation, or the removal of less than 12 lymph nodes (Vodenkova et al., 2020). The typical approach for treating rectal cancer involves using radiation therapy and combining it with simultaneous fluoropyrimidines (FPs). When dealing with metastatic disease, treatments aimed at stopping the angiogenesis, like bevacizumab, ramucirumab, and aflibercept, are often given alongside fluoropyrimidine-based chemotherapy. Epidermal Growth Factor Receptor (EGFR) inhibitors, including panitumumab and cetuximab, are also frequently used. In advanced stages of metastatic CRC (mCRC), regorafenib (a tyrosine kinase inhibitor that targets blood vessels) and trifluridine-tipiracil (a type of fluoropyrimidine) have displayed limited effectiveness (Vodenkova et al., 2020). In this section, we review a diversity of studies on sensitizing CRC cells to the mentioned drugs through miR-143 (these studies are also summarized in Table 2).

**TABLE 2:** A summary of studies investigating the relation between mir-143 and drug resistance in colorectal cancer cells.

Drug	Model	Cell line/Animal	Result(s) (mechanism of sensitization)	Reference
5-Fluorouracil (5-FU)	*In vitro*/*Ex vivo*	5-FU-R cells	• Re-sensitizes by targeting Hexokinase 2 (HK II) and inhibiting glycolysis	Chen et al. (2023)
5-Fluorouracil (5-FU)	*In vitro*	HCT116 cells	• Increases apoptosis by inhibiting the ERK5/NF-κB pathway	Borralho et al. (2009)
Capecitabine	Human study	mCRC patients	• Contradictory: Low miR-143 correlated with better response (longer PFS)	Simmer et al. (2015)
Bevacizumab	*Ex vivo*	mCRC patient biopsies	• Correlational: High miR-143 correlated with longer PFS	Romero-Lorca et al. (2021)
Cetuximab	*In vitro*	HCT116 & SW480 cells	• Sensitizes KRAS-mutant & wild-type cells • Mechanism: Enhances ADCC (not direct pathway)	Gomes et al. (2016)
Oxaliplatin	*In vitro*	HCT116, HT29etc.	• Sensitizes by directly targeting IGF1R	Qian et al. (2013)
Oxaliplatin	*In vitro*	HCT116 cells	• Sensitizes by increasing intracellular ROS (via SOD1 suppression)	Gomes et al. (2018)
Paclitaxel	*In vitro*	KRAS-mutant (LoVo)	• Re-sensitizes KRAS-mutant cells by targeting the KRAS pathway	Fei et al. (2016)

### 5-Fluorouracil (5-FU)

5-Fluorouracil (5-FU) is an antimetabolite that has been a cornerstone of CRC chemotherapy for decades. It functions by inhibiting thymidylate synthase, thereby disrupting DNA synthesis. Overcoming 5-FU resistance is a critical clinical goal.

miR-143 appears to re-sensitize CRC cells to 5-FU via at least two primary signaling cascades.

1. Metabolic Reprogramming: The first mechanism involves the direct targeting of Hexokinase 2 (HK II), a key enzyme in the glycolytic pathway. By inhibiting HK II, miR-143 interferes with the enhanced glucose metabolism (Warburg effect) that 5-FU-resistant cells depend on, effectively starving them of energy and restoring sensitivity (Chen et al., 2023).
2. Pro-Survival Signaling: The second mechanism involves the suppression of the ERK5/NF-κB signaling pathway. Inhibition of this cascade by miR-143 leads to a reduction in anti-apoptotic proteins like Bcl-2 and a subsequent increase in the activity of caspase-3, -8, and -9, thereby restoring the cell’s ability to undergo apoptosis in response to 5-FU treatment (Borralho et al., 2009).

### Capecitabine

The link between miR-143 and capecitabine, a 5-FU pro-drug, is less clear and presents a mechanistic contradiction. A study by Simmer et al. (2015) found that *lower* miR-143 levels correlated with a *better* response (longer PFS). This suggests a complex, context-dependent role. The precise signaling cascade is unelucidated, but this finding indicates miR-143 might, in this specific context, interfere with the metabolic activation of capecitabine.

### Bevacizumab

Similarly, the link to bevacizumab, a monoclonal antibody that inhibits VEGF, is currently correlational. High miR-143 expression was linked to longer PFS in patients receiving bevacizumab (Romero-Lorca et al., 2021), suggesting it modulates the anti-angiogenic response. However, the specific signaling cascade by which miR-143 interacts with the VEGF pathway or tumor microenvironment to achieve this sensitization is not yet defined.

### Cetuximab

miR-143s role in sensitizing cells to cetuximab, an EGFR inhibitor, is primarily immune-mediated, rather than a direct inhibition of the EGFR pathway itself. Overexpression of miR-143 was found to significantly enhance antibody-dependent cellular cytotoxicity (ADCC), a key mechanism of cetuximab. This enhanced ADCC, which was observed regardless of the tumor’s KRAS mutation status, is linked to a restoration of the apoptotic cascade, evidenced by a rise in caspase-3/7 activity and a reduction in the anti-apoptotic protein Bcl-2 (Gomes et al., 2016).

### Oxaliplatin

Sensitization to oxaliplatin, a platinum-based agent, appears to operate through two distinct mechanisms:

1. IGF1R Pathway Inhibition: miR-143 directly targets and suppresses the Insulin-like Growth Factor 1 Receptor (IGF1R). By inhibiting the pro-survival signaling that flows from this receptor, miR-143 enhances the DNA-damaging efficacy of oxaliplatin (Qian et al., 2013).
2. Oxidative Stress Modulation: miR-143 overexpression leads to a surge in intracellular Reactive Oxygen Species (ROS), partly by suppressing antioxidant enzymes like SOD1. This high-ROS environment, induced by miR-143, pushes the cancer cells past a critical apoptotic threshold when combined with the additional oxidative stress from oxaliplatin treatment (Gomes et al., 2018).

### Paclitaxel

For paclitaxel, the mechanism is directly tied to the central KRAS oncogenic pathway. Fei et al. (2016) demonstrated that miR-143 mimics could re-sensitize KRAS-mutant CRC cells to treatment. The signaling cascade inhibited is the mutant KRAS pathway itself. By suppressing its expression, miR-143 reverses the metastatic characteristics and restores the apoptotic sensitivity that KRAS mutations normally block, allowing paclitaxel to function effectively.

The role of miR-143 as a drug sensitizer is best understood when compared to other well-studied miRNAs in CRC. For instance, miR-21, a prominent *onco-miR*, has the opposite effect: its high expression is strongly correlated with *resistance* to 5-FU and oxaliplatin by suppressing apoptotic pathways (Valeri et al., 2010). In contrast, miR-34a, like miR-143, is a well-known tumor suppressor that sensitizes CRC cells to 5-FU and oxaliplatin, primarily by acting downstream of p53 to induce apoptosis (Wu et al., 2011). The unique position of miR-143 stems from its specific, potent regulation of pathways that other tumor-suppressor miRNAs do not target as directly. Its ability to simultaneously inhibit the KRAS signaling axis, reverse EMT, and shut down metabolic reprogramming (by targeting HK II) places it at a critical nexus. This makes miR-143 a particularly valuable candidate, as it not only sensitizes cells to traditional chemotherapy but also shows potential in overcoming resistance to targeted therapies in KRAS-mutant tumors, a major clinical challenge.

### The clinical trial landscape for miR-143

Despite the compelling preclinical evidence, the translation of miR-143-based therapeutics into clinical practice remains in its infancy. A search of clinical trial registries (e.g., ClinicalTrials.gov) reveals that the majority of studies involving miR-143 in CRC are observational or prognostic. These trials primarily aim to validate miR-143s role as a diagnostic or prognostic biomarker by correlating its expression levels (in tissue or liquid biopsies) with disease stage, progression-free survival (PFS), or response to standard-of-care chemotherapy.

To date, interventional trials—those that actively administer a miR-143 mimic (a synthetic version of the miRNA) as a therapeutic agent—are lacking for CRC. The primary hurdles, as discussed in the limitations, include systemic delivery, stability, and off-target effects. While trials for other miRNA mimics (such as mimics of miR-34) have entered Phase I for other cancers, the therapeutic application of miR-143 for CRC is still firmly in the preclinical phase, underscoring the gap that must be bridged between laboratory findings and patient application, a challenge primarily centered on the delivery and stability issues discussed in the following section.

### Delivery strategies for miR-143 therapeutics

A primary challenge in translating miR-143 into a clinical therapeutic is effective and safe delivery. Because naked RNA molecules are quickly degraded by nucleases in the bloodstream and are poorly taken up by cells, sophisticated delivery vectors are essential (68). The ideal system must protect the miR-143 mimic, ensure its stability in circulation, and facilitate its specific uptake by CRC cells while avoiding healthy tissues.

### Nanocarrier systems

Nanocarrier-based systems represent the most widely explored and clinically advanced strategy. These are broadly categorized into lipid-based, polymeric, and inorganic nanoparticles. Lipid-based nanoparticles (LNPs), including liposomes, are highly favored. Their lipophilic nature allows them to easily encapsulate the negatively charged miR-143 mimic, protect it from degradation, and efficiently fuse with the cell membrane to release their cargo. Their design is highly tunable, and they have been clinically validated (e.g., for siRNA and mRNA vaccines) (O’Neill and Dwyer, 2020). Polymeric nanoparticles, using biodegradable materials like poly (lactic-co-glycolic acid) (PLGA), offer advantages such as high stability and the potential for sustained, controlled release of the miR-143 mimic over time. Inorganic nanoparticles, such as gold nanoparticles (AuNPs), offer a rigid scaffold that is easily functionalized with targeting ligands (e.g., antibodies against CRC-specific receptors) to improve tumor-specific accumulation (Sousa and Conde, 2022).

### Exosome-mediated delivery

Exosomes, which are natural, cell-derived nanovesicles (30–150 nm), are being investigated as a novel “biologic” delivery platform. They are essentially nature’s own system for intercellular communication, adept at transferring biological cargo, including endogenous miRNAs. Their primary advantages include high biocompatibility, low immunogenicity, and an inherent ability to cross biological barriers and be taken up by recipient cells (Di et al., 2017). The challenge, however, lies in efficiently loading therapeutic quantities of exogenous miR-143 mimics into them (a process called ‘loading’) and scaling up their production for clinical use. Nonetheless, their “natural” targeting properties make them a highly promising avenue for development.

### Viral vectors

Viral vectors, such as adeno-associated virus (AAV) or lentivirus, are the most efficient systems for gene delivery due to their natural ability to infect cells and deliver genetic material. These vectors are typically engineered to express a short-hairpin RNA (shRNA) that is then processed by the cell’s own machinery into mature miR-143. This approach can lead to stable, long-term expression of the miRNA, which may be beneficial (Holjencin and Jakymiw, 2022). However, this strategy faces significant hurdles for miRNA therapy, including (Bray et al., 2018): Immunogenicity, where the host immune system may attack the viral vector (Dekker et al., 2019); Insertional mutagenesis, the risk that the viral DNA integrates into the host genome and disrupts a critical gene; and (Li et al., 2021) Complex manufacturing and high costs. Given that miRNA mimics are often intended as transient drugs, the risks associated with permanent gene-modifying vectors are often considered too high for this application.

### miR-143 as a predictive biomarker for therapeutic response

Beyond its general prognostic value, miR-143 expression levels hold significant potential as a predictive biomarker to guide patient stratification and treatment selection. A predictive biomarker provides information on the likely benefit from a *specific* therapy. The preclinical evidence strongly suggests that miR-143 status could be used to predict which patients will respond to, or develop resistance to, standard-of-care agents.

For example, a low level of miR-143 in a patient’s tumor biopsy could serve as a predictive marker for:

- Potential resistance to 5-FU and oxaliplatin: Low miR-143 correlates with high expression of its targets, such as HK II and IGF1R, which are known mechanisms of resistance. These patients might benefit from alternative first-line agents or the co-administration of a miR-143 mimic.
- Likely non-response to cetuximab: In cases where cetuximab’s efficacy relies on ADCC, low miR-143 might predict a poor immune-mediated response.

Conversely, and more perplexingly, the finding by Simmer et al. (2015) suggests that low miR-143 levels might predict a *better* response to capecitabine.

This highlights the critical need for validation studies. If confirmed, testing for miR-143 expression could become a routine part of the molecular workup for CRC, alongside *KRAS* and *BRAF* mutational analysis, to help oncologists tailor therapeutic strategies based on the tumor’s specific molecular vulnerabilities.

The strongest evidence for miR-143s therapeutic potential lies in its ability to re-sensitize resistant cells through clear metabolic (targeting HK II) and oxidative stress (targeting SOD1/IGF1R) pathways. A major gap, however, exists between these compelling *in vitro* findings and clinical application. The majority of these studies are performed in 2D cell cultures, and it remains unknown if miR-143 mimics can achieve sufficient concentration *in vivo* to overcome the complex, multifactorial resistance mechanisms present in a heterogeneous human tumor.

### Limitations and challenges in clinical translation

Notwithstanding the considerable progress in elucidating the role of miR-143 in CRC, numerous constraints impede its prompt clinical application. A balanced outlook requires a critical discussion of the significant safety, efficacy, and translational hurdles that remain.

### Safety and immunogenicity

A primary safety concern for any RNA-based therapeutic is immunogenicity. The introduction of exogenous, double-stranded RNA mimics can be recognized by the innate immune system (e.g., via Toll-like receptors), potentially triggering a systemic inflammatory response, cytokine release, and nonspecific toxicity. This immune activation must be carefully evaluated and mitigated, often by chemical modifications to the RNA backbone.

### Off-target effects

This remains one of the most significant challenges. A single miRNA has a short seed sequence (6-8 nucleotides), which allows it to bind with partial complementarity to hundreds of different mRNA targets. Flooding a cell with a high, non-physiological concentration of a miR-143 mimic could lead to the unintended suppression of essential “housekeeping” genes or other tumor suppressors, resulting in unpredictable off-target toxicity in both cancerous and healthy tissues.

### Challenges in clinical translation

The “delivery problem” is the central hurdle in clinical translation. As discussed, naked miRNAs are rapidly degraded by serum nucleases. While delivery vectors are a solution, they present their own challenges:

- Biodistribution: Many nanocarriers are non-specifically cleared by the reticuloendothelial system (i.e., the liver and spleen), leading to low accumulation in the tumor and potential off-target toxicity in these organs.
- Tumor Penetration: Solid CRC tumors are dense and have high interstitial fluid pressure, making it difficult for nanocarriers to penetrate deep into the tumor mass and reach all cancer cells.
- Targeting: Achieving true, active targeting *only* to CRC cells while sparing healthy intestinal epithelium is exceptionally difficult.

Finally, biological and logistical barriers include the profound heterogeneity of CRC tumors, meaning not all cells may be responsive to miR-143. Furthermore, the variability in miR-143 data across studies (due to non-standardized detection methods) complicates its validation as a reliable biomarker, and the high cost of manufacturing clinical-grade RNA therapeutics remains a significant barrier to widespread adoption.

## Conclusions and key messages

miR-143 has been identified as a critical tumor suppressor in CRC, exerting a range of regulatory influences on the processes of cancer initiation, progression, and response to therapeutic interventions. It is instrumental in the modulation of significant oncogenic signaling pathways and the Wnt/β-catenin signaling cascade, while also impacting various biological processes such as epithelial-mesenchymal transition, cell cycle regulation, apoptosis, invasiveness, and angiogenesis. The persistent downregulation of miR-143 observed in CRC tissues and samples obtained from patients underscores its potential utility as both a diagnostic and prognostic biomarker. Furthermore, an increasing body of evidence substantiates its role in forecasting and influencing resistance to standard chemotherapeutic treatments and targeted therapies, thereby positioning it as a promising candidate for enhancing therapeutic efficacy and addressing drug resistance issues. Notwithstanding its significance, miR-143 is frequently underrepresented in contemporary clinical protocols and biomarker panels, with the majority of existing reviews lacking a concentrated, integrative, and mechanistically grounded examination of its varied functions in CRC. This comprehensive review addresses a significant void by methodically examining the molecular roles of miR-143, its ramifications for diagnostic and prognostic applications, its regulatory impact on drug resistance, and the innovative therapeutic strategies developed to either restore or deliver its functionality. Recent advancements in the delivery mechanisms for miRNAs—including nanoparticle-mediated vectors, exosomes, and viral vectors—present translational prospects for leveraging miR-143 as both a therapeutic and predictive biomarker within the realm of precision oncology. Ultimately, the incorporation of miR-143 into the management of CRC may unveil a novel paradigm in personalized medicine, thereby enabling healthcare practitioners to stratify patients according to their molecular profiles, customize treatment regimens, and forecast resistance outcomes. The amalgamation of fundamental research, biomarker identification, and therapeutic advancements surrounding miR-143 highlights its potential clinical significance. Subsequent validation through clinical trials, refinement of delivery methodologies, and standardization of detection protocols are imperative subsequent actions to transition miR-143 from laboratory research to clinical application.

## Future perspectives: integrating miR-143 into personalized oncology

To facilitate the integration of miR-143 into personalized oncology frameworks for CRC, several pivotal avenues warrant exploration. Firstly, extensive clinical trials are imperative to substantiate the diagnostic and prognostic significance of miR-143 across a heterogeneous array of patient cohorts and CRC classifications. The standardization of detection methodologies—such as quantitative reverse transcription polymerase chain reaction, next-generation sequencing, and liquid biopsy technologies—remains crucial to guarantee reproducibility, sensitivity, and clinical dependability. Concurrently, mechanistic investigations ought to persist in elucidating the context-specific functions of miR-143, encompassing its interactions with long non-coding RNAs, circular RNAs, and additional constituents of the tumor microenvironment. A comprehensive understanding of the upstream epigenetic and transcriptional regulators of miR-143 will further illuminate its dynamic involvement in CRC progression and the phenomenon of drug resistance. Another pivotal pathway pertains to the innovation of precision-medicine combinations. While enhancing delivery systems (nanoparticles, exosomes) is critical, the true therapeutic potential of miR-143 may lie in synergistic strategies. The integration of miR-143 mimics with established chemotherapeutic agents (like 5-FU or oxaliplatin) or targeted therapies (like cetuximab) could lead to enhanced outcomes and reverse drug resistance. A particularly promising frontier is combining miR-143 therapeutics with immunotherapy, such as immune checkpoint inhibitors (e.g., anti-PD-1/PD-L1). Given that miR-143 can modulate oncogenic pathways (like KRAS) and enhance ADCC, it is plausible that its restoration could sensitize “cold” tumors to immune attack, thereby augmenting the efficacy of checkpoint blockade. Preclinical models, particularly patient-derived organoids and xenografts, must be used to evaluate the safety and therapeutic synergy of these novel combination interventions *in vivo*.
